# Supplementary material for: The genetic basis of dermatophytosis skin infection susceptibility
Source: Nat Commun. 2026 Mar 6;17:3554. doi: 10.1038/s41467-026-69670-z (PMC13087286; doi:10.1038/s41467-026-69670-z)
Supplement: Supplementary file 1 — Supplementary Information [file 41467_2026_69670_MOESM1_ESM.pdf]

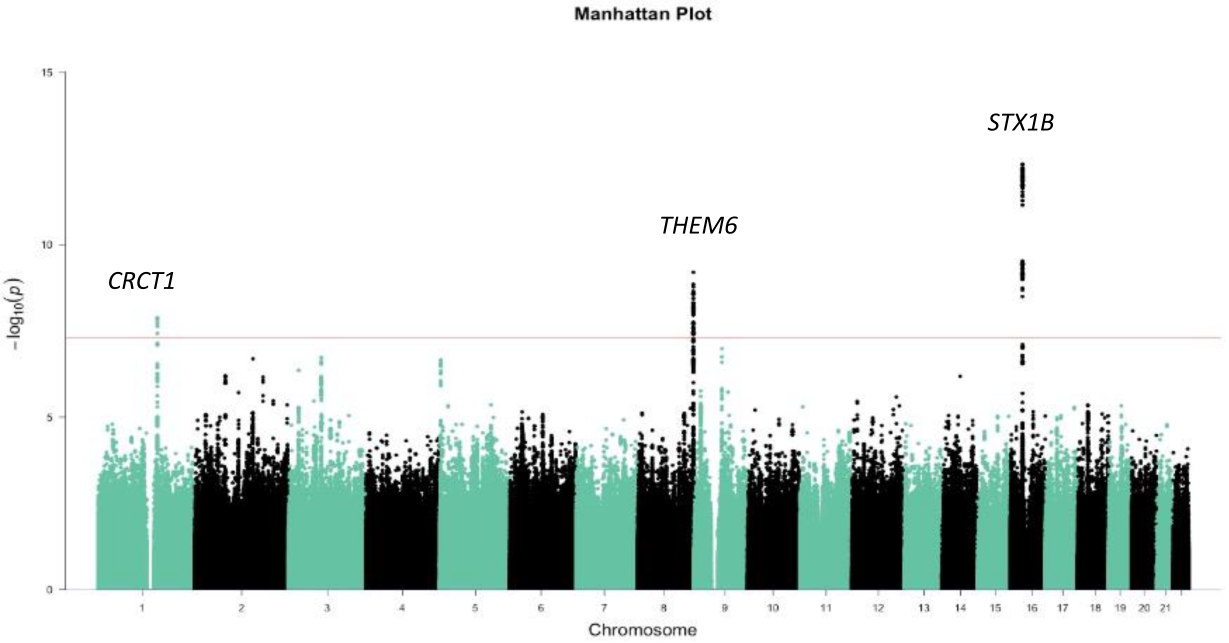

Supplementary figure 1. Manhattan plot for FinnGen dermatophytosis GWAS.

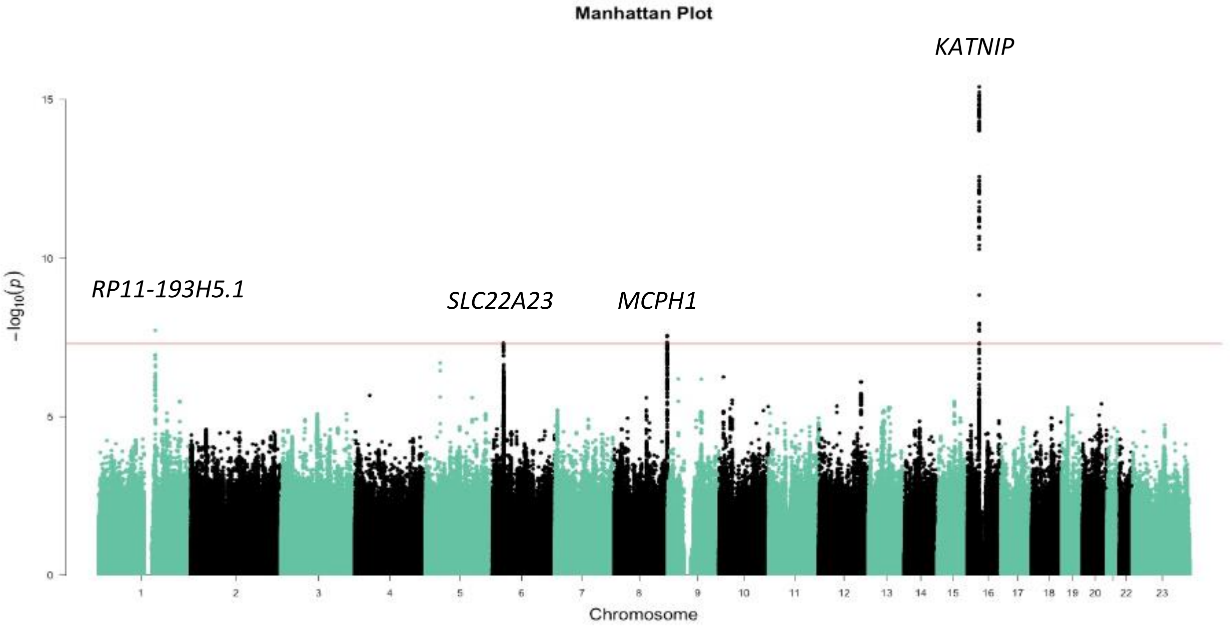

Supplementary figure 2. Manhattan plot for UK biobank dermatophytosis GWAS.

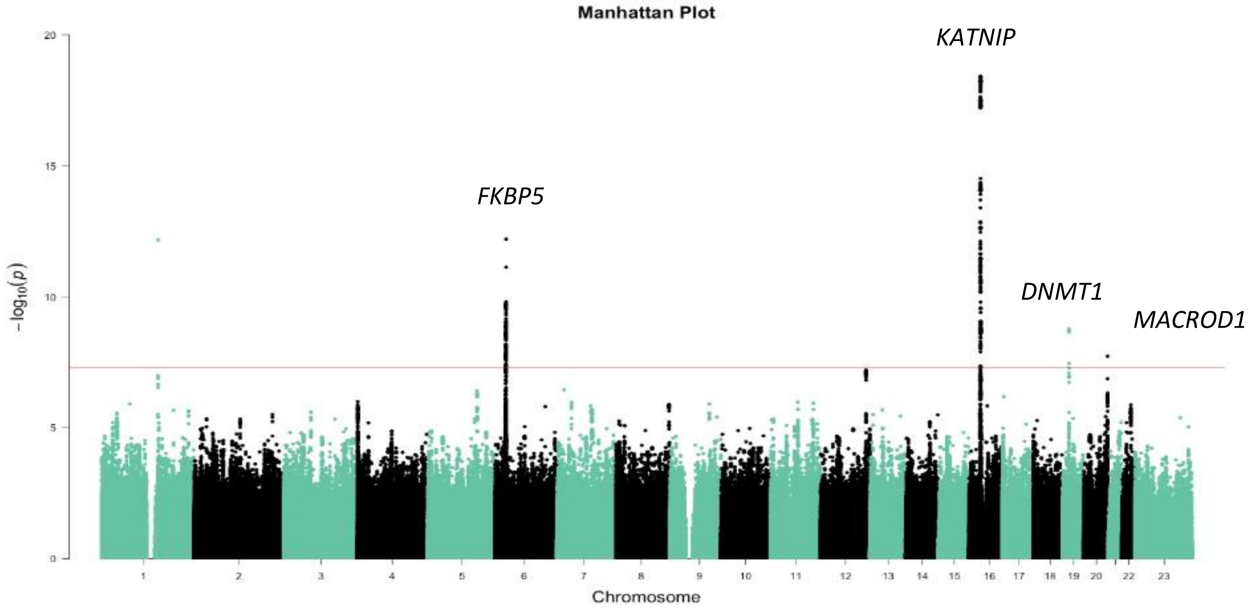

Supplementary figure 3. Manhattan plot for Estonian biobank dermatophytosis GWAS.

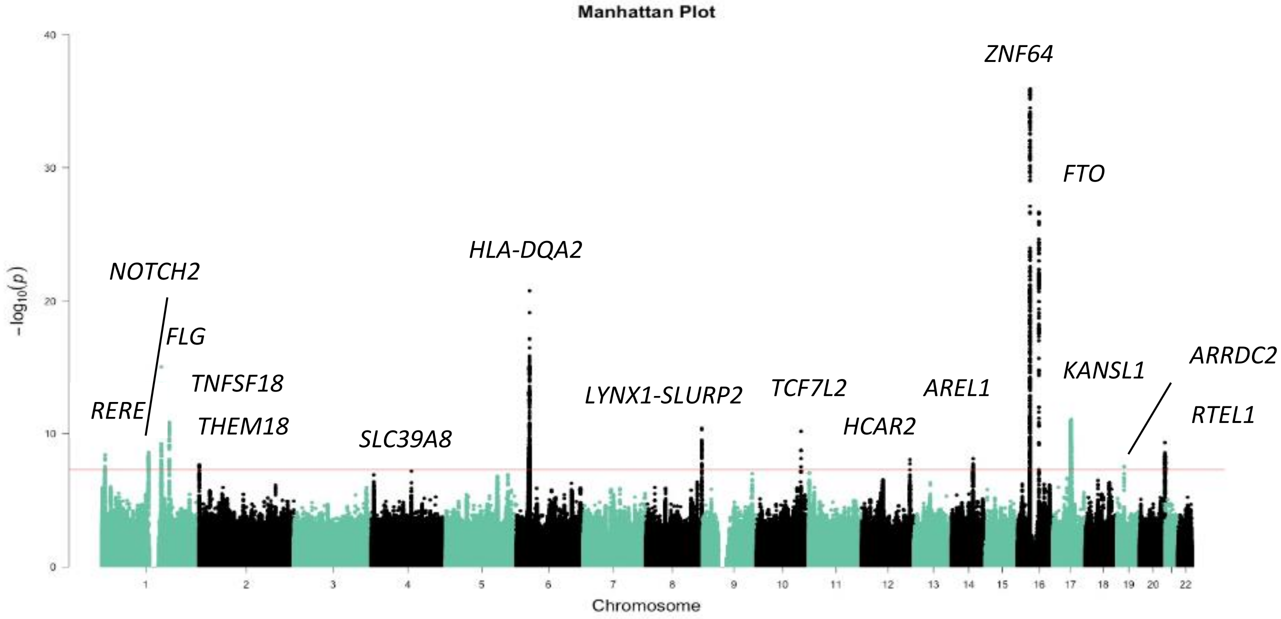

Supplementary figure 4. Manhattan plot for MVP dermatophytosis GWAS.

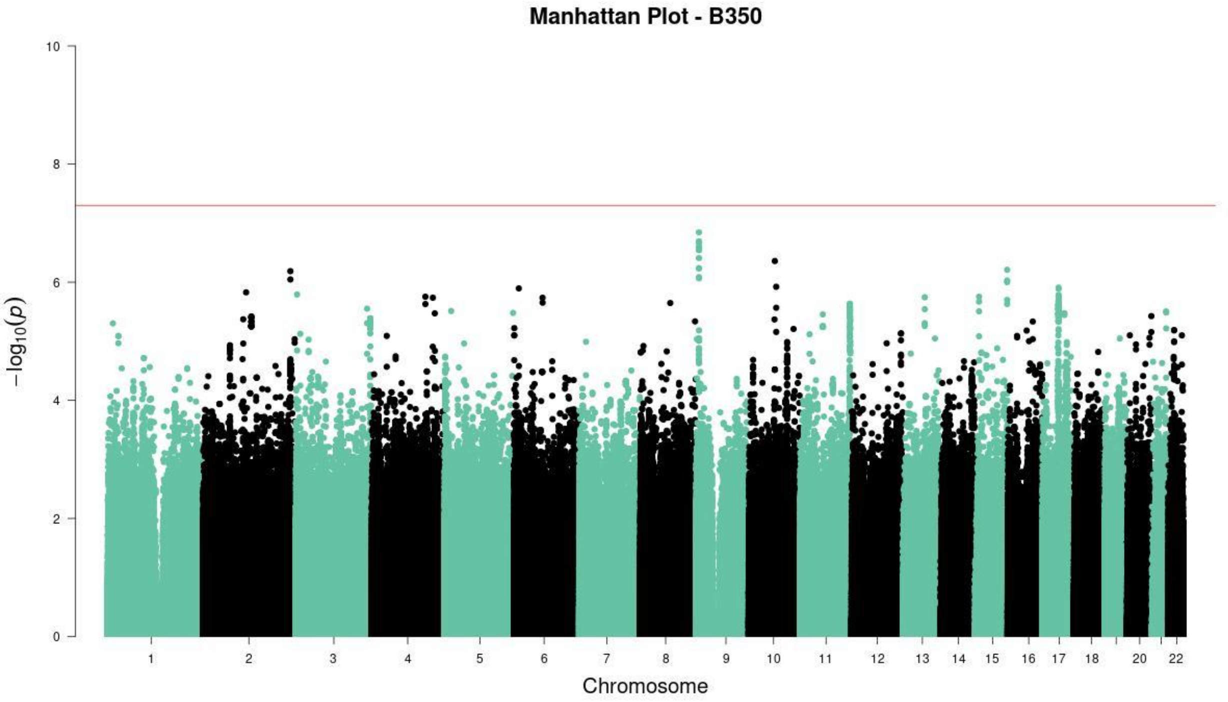

Supplementary figure 5. Manhattan plot for B35.0 dermatophytosis GWAS meta-analysis using FinnGen and Estonian biobank.

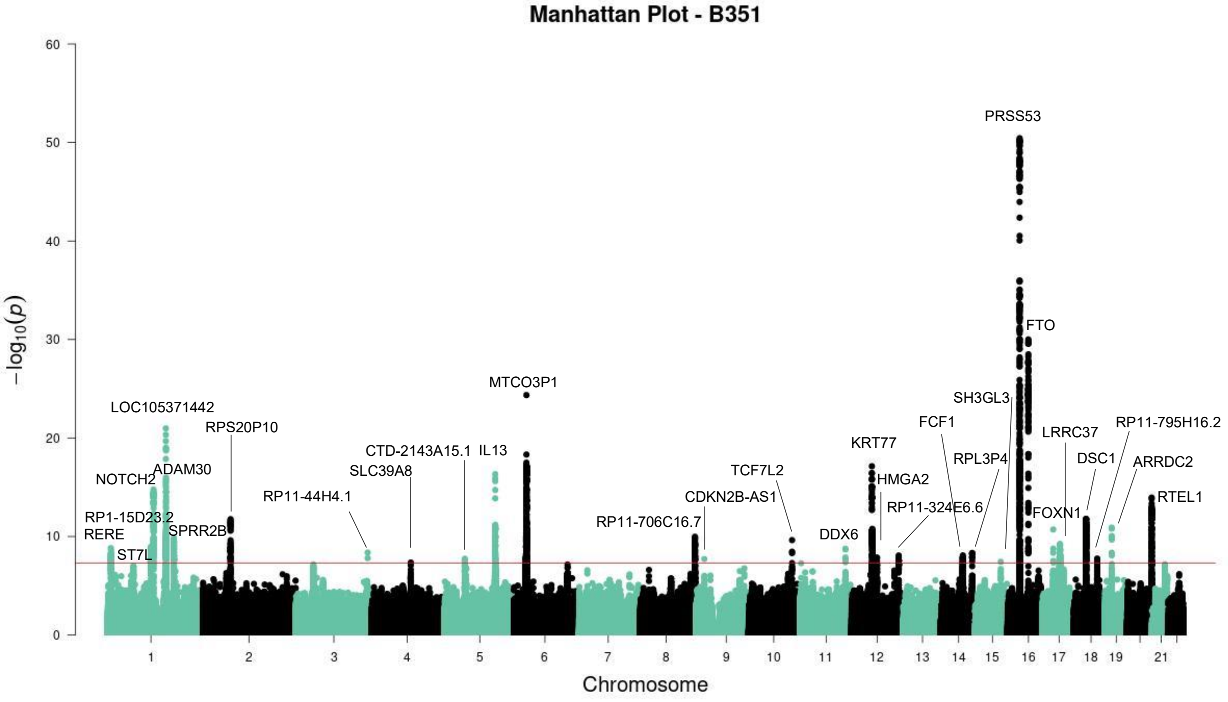

Supplementary figure 6. Manhattan plot for B35.1 dermatophytosis GWAS meta-analysis using FinnGen, Estonian biobank, UK biobank, and MVP.

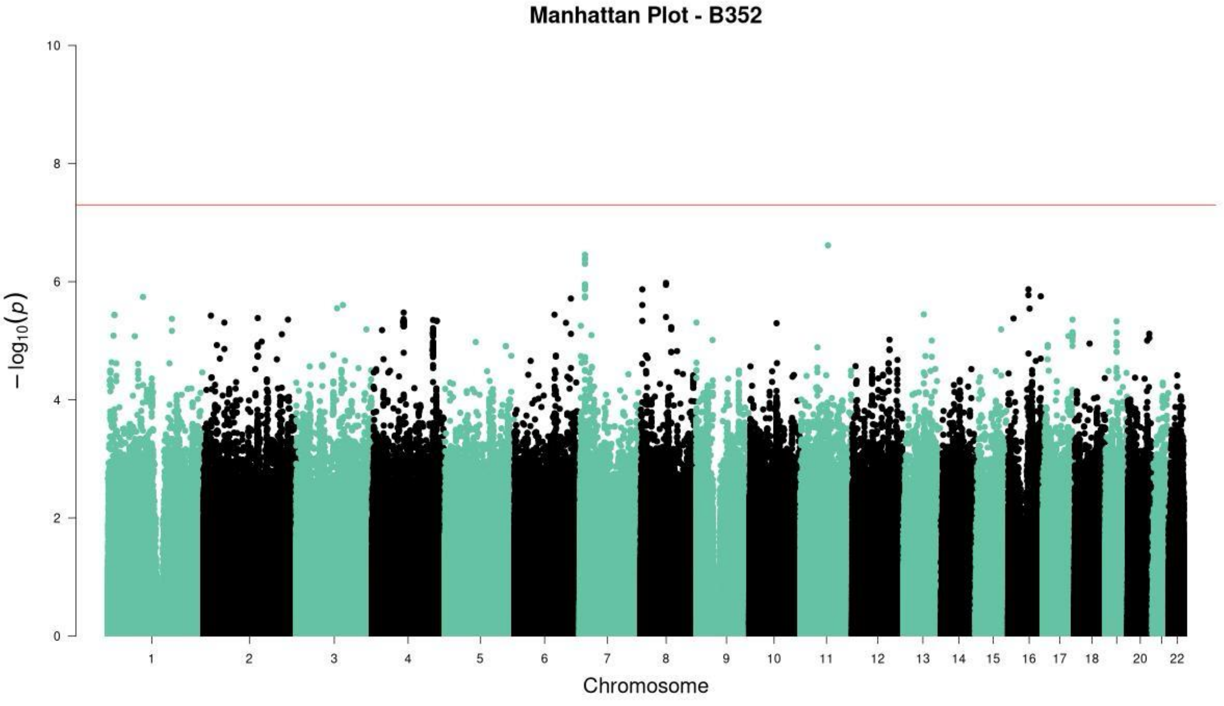

Supplementary figure 7. Manhattan plot for B35.2 dermatophytosis GWAS meta-analysis using FinnGen, Estonian biobank and UK biobank.

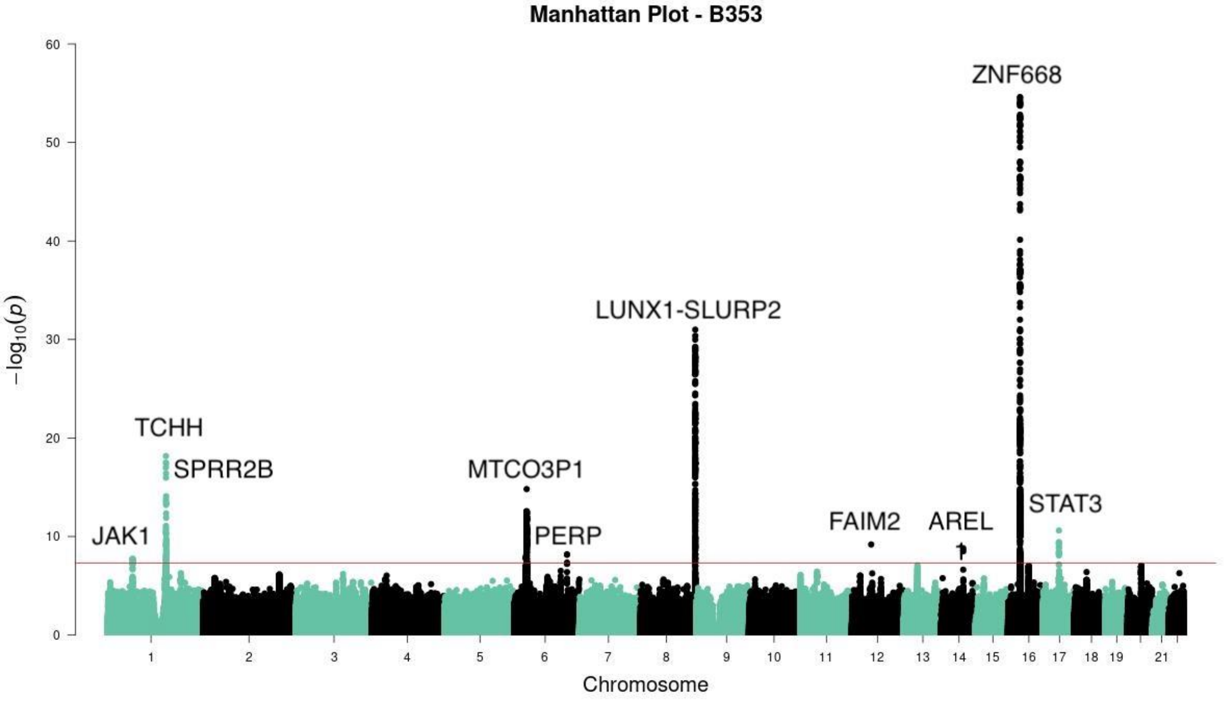

Supplementary figure 8. Manhattan plot for B35.3 dermatophytosis GWAS meta-analysis using FinnGen, Estonian biobank, UK biobank, and MVP.

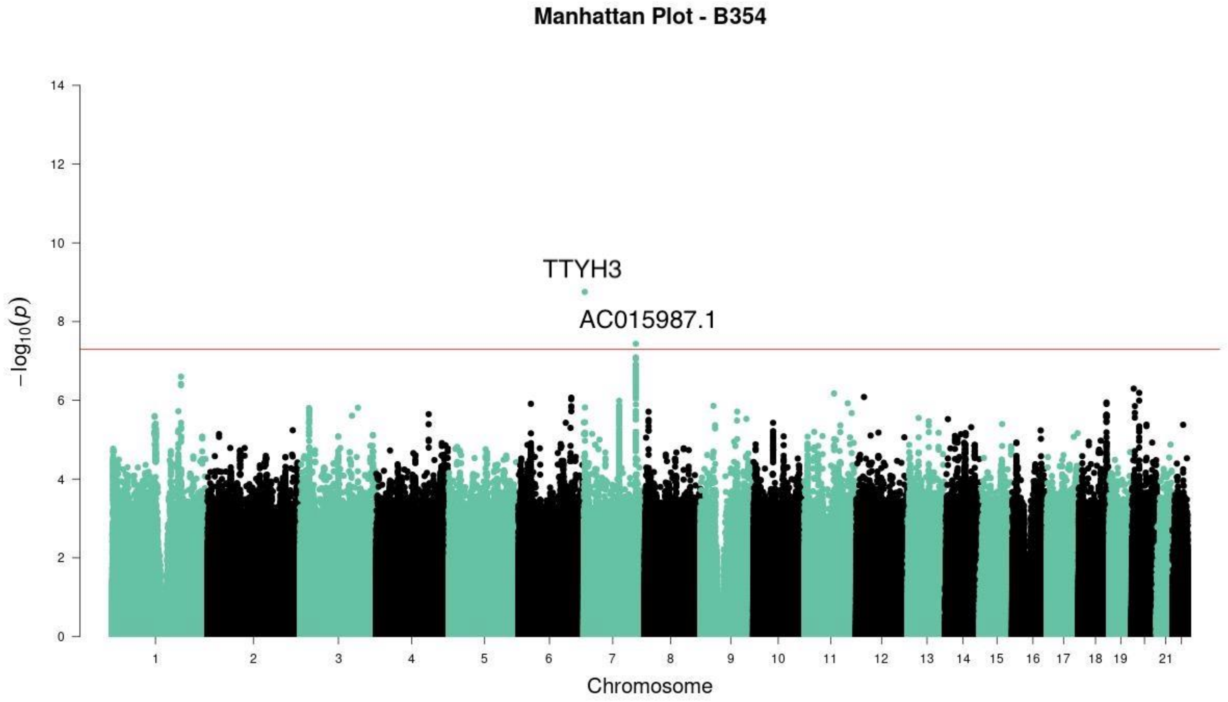

Supplementary figure 9. Manhattan plot for B35.4 dermatophytosis GWAS meta-analysis using FinnGen, Estonian biobank, UK biobank, and MVP.

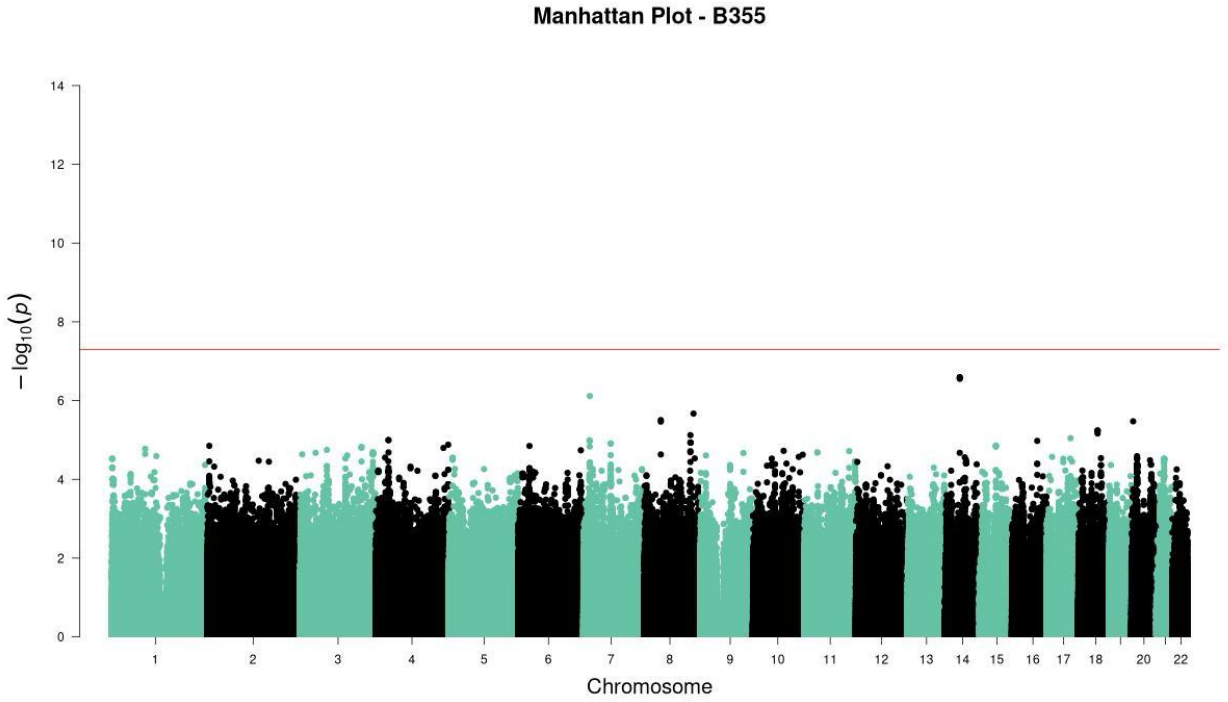

Supplementary figure 10. Manhattan plot for B35.5 dermatophytosis GWAS meta-analysis using FinnGen and Estonian biobank.

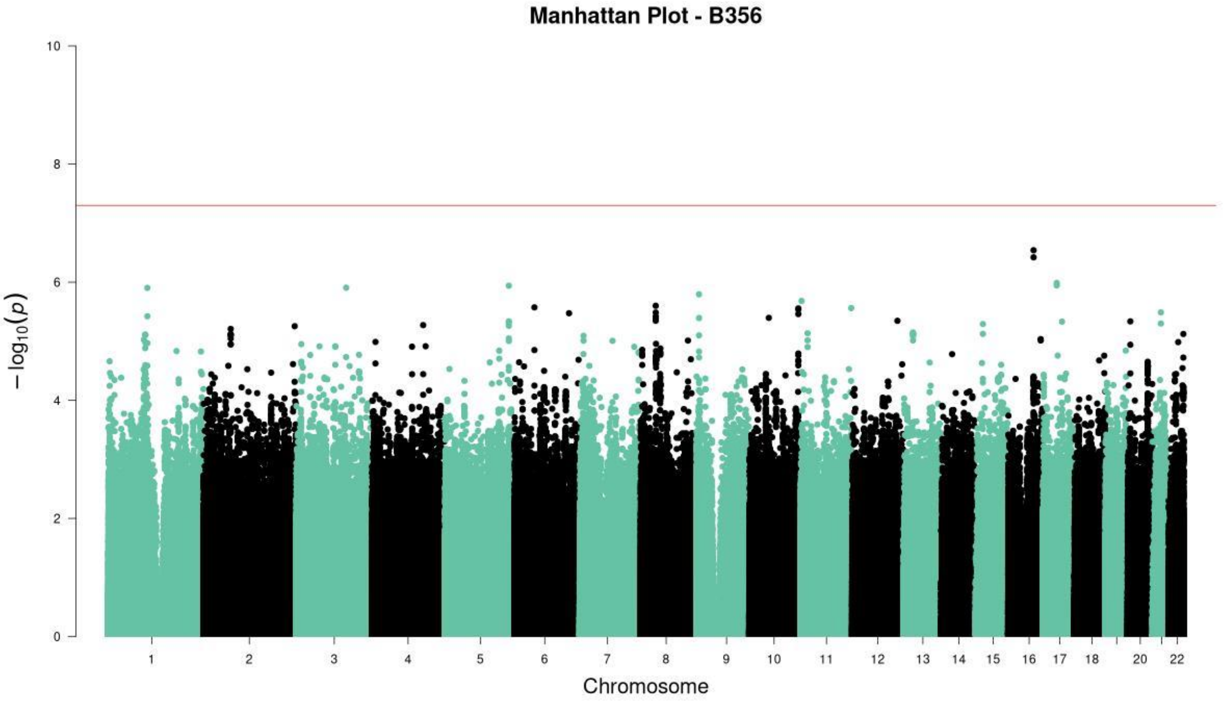

Supplementary figure 11. Manhattan plot for B35.6 dermatophytosis GWAS meta-analysis using FinnGen, Estonian biobank and UK biobank.

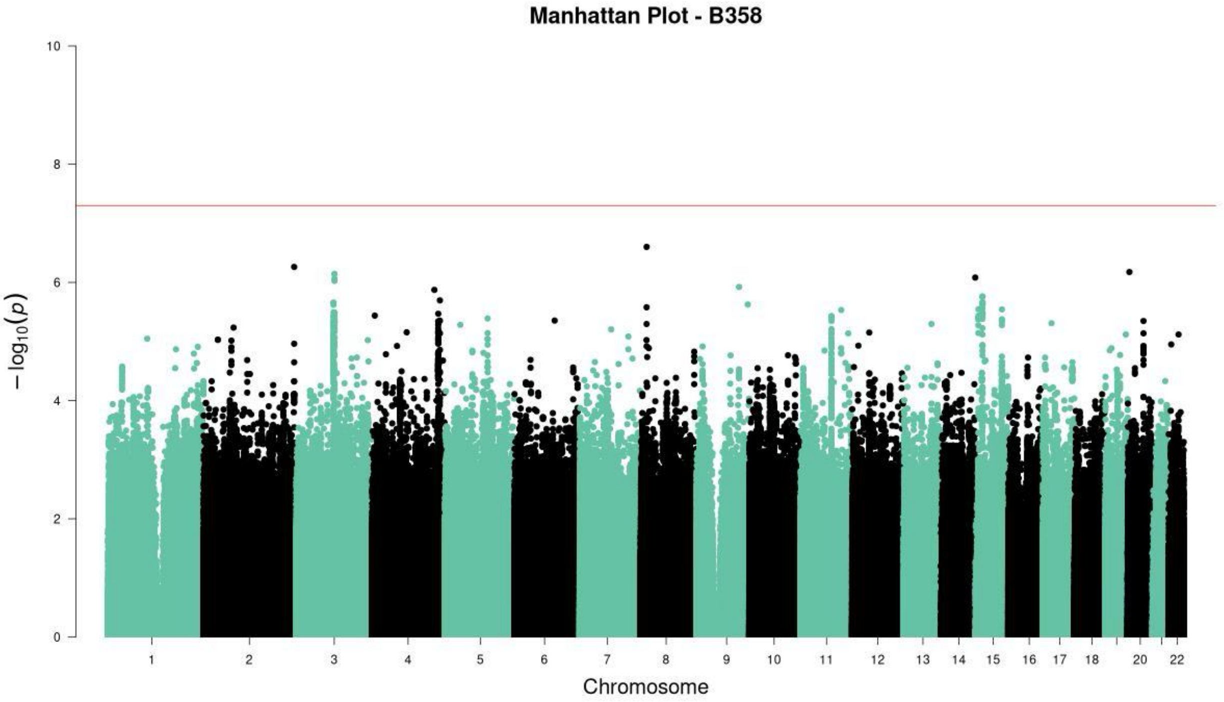

Supplementary figure 12. Manhattan plot for B35.8 dermatophytosis GWAS meta-analysis using FinnGen, Estonian biobank and UK biobank.

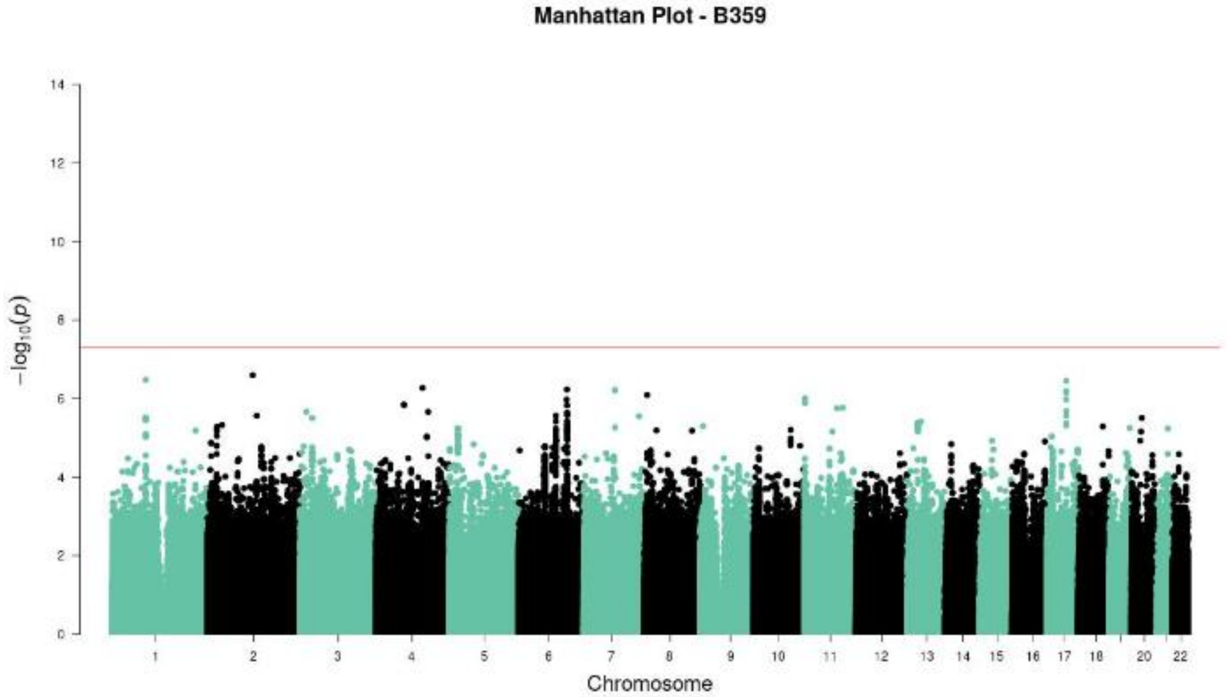

Supplementary figure 13. Manhattan plot for B35.9 dermatophytosis GWAS meta-analysis using FinnGen and UK biobank.
