# Supplementary material for: The genetic basis of dermatophytosis skin infection susceptibility
Source: Nat Commun. 2026 Mar 6;17:3554. doi: 10.1038/s41467-026-69670-z (PMC13087286; doi:10.1038/s41467-026-69670-z)
Supplement: Supplementary file 2 — Description of Additional Supplementary Files [file 41467_2026_69670_MOESM2_ESM.pdf]

Description of Additional Supplementary Files

Supplementary Data S1. Dermatophytosis lead variants, their association statistics in meta-analysis and individual cohorts, and possible consequences. Association statistics in GWAS are based on two-sided Wald tests under an additive genetic model. No multiple testing correction has been applied for the data, hence using 10E-8 as the significance threshold.

Supplementary Data S2. Colocalization analysis results for SLURP2 lead variant. PP.HX.abf represent the posterior probability of the approximate Bayes factor for different hypotheses: H0 (no causal variant identified in either dataset), H1 (causal variant identified in trait 1 only - Dermatophytosis), H2 (causal variant identified in differential expression in the tissue), H3 (two distinct causal variants, one for Dermatophytosis and one for the differential expression in the tissue), and H4 (one common causal variant for both Dermatophytosis and the differential expression in the tissue).

Supplementary Data S3. Colocalization analysis results for FTO lead variant. PP.HX.abf represent the posterior probability of the approximate Bayes factor for different hypotheses: H0 (no causal variant identified in either dataset), H1 (causal variant identified in trait 1 only - Dermatophytosis), H2 (causal variant identified in differential expression in the tissue), H3 (two distinct causal variants, one for Dermatophytosis and one for the differential expression in the tissue), and H4 (one common causal variant for both Dermatophytosis and the differential expression in the tissue).

Supplementary Data S4. Narrow tissue LDSC analysis for dermatophytosis identities connective/bone tissue and immune cells relevant to dermatophytosis. Enrichment p-values were derived from a two-sided Z-test of the null hypothesis that the regression coefficient for each annotation equals zero. No multiple hypothesis correction has been applied to p-values but Bonferroni corrected threshold is used to assess significance.

Supplementary Data S5. Heritability analysis using sLDSC analysis. Table shows the partitioned heritability of Dermatophytosis in skin-related cell types. Coefficient p-values were derived from a two-sided Z-test of the null hypothesis that the regression coefficient for each annotation equals zero. No multiple hypothesis correction has been applied to p-values as Bonferroni corrected threshold is used to assess significance.

Supplementary Data S6. Heritability analysis using sLDSC analysis. Table shows the partitioned heritability of Dermatophytosis in immune-related cell types. Coefficient p-

values were derived from a two-sided Z-test of the null hypothesis that the regression coefficient for each annotation equals zero. No multiple hypothesis correction has been applied to p-values as Bonferroni corrected threshold is used to assess significance.

Supplementary Data S7. Genetic correlation analysis results. Table shows the genetic correlation between Dermatophytosis and other skin diseases, infections, obesity and BMI. P-values were derived from a two-sided Z-test of the null hypothesis that the regression coefficient for each annotation equals zero. No multiple hypothesis correction has been applied to p-values.

Supplementary Data S8. Case and controls used for running the GWAS of dermatophytosis subtypes using FinnGen, EstBB, UKB, and MVP.

Supplementary Data S9. The meta-analysis using FinnGen, EstBB, UKB, and MVP is performed for each dermatophytosis subtype. The table shows the association statistics of the subtypes for the main lead variants in the original meta-analysis. The lead variants from main meta-analysis that replicate in the subtype analysis are shown in bold ($p < 0.05$).

Supplementary Data S10. lead variants for the dermatophytosis subtypes meta analysis.
